# MiR-21 attenuates FAS-mediated cardiomyocyte apoptosis by regulating HIPK3 expression

**DOI:** 10.1042/BSR20230014

**Published:** 2023-09-05

**Authors:** Xinyu Wang, Tingting Zhang, Jianlong Zhai, Zhongli Wang, Yan Wang, Lili He, Sai Ma, Hanying Xing, Yifang Guo

**Affiliations:** 1 College of Postgraduate, Hebei North University, Zhangjiakou, Hebei, China; 2 Department of Geriatric Cardiology, Hebei General Hospital, Shijiazhuang, Hebei, China; 3 Department of Cardiology, Hebei General Hospital, Shijiazhuang, Hebei, China; 4 Department of Physical Examination Center, Hebei General Hospital, Shijiazhuang, Hebei, China; 5 Department of Internal Medicine, Hebei General Hospital, Shijiazhuang, China; 6 Hebei Key Laboratory of Metabolic Disease, Hebei General Hospital, Shijiazhuang, Hebei, China

**Keywords:** apoptosis, cardiomyocytes, hypoxia, microRNA

## Abstract

MicroRNA-21 (miR‐21) plays an anti-apoptotic role following ischemia–reperfusion (I/R) injury (IRI) *in vivo*; however, its underlying mechanism remains unclear. The present study explored the effects of miR-21 and homeodomain interacting protein kinase 3 (HIPK3) on cardiomyocyte apoptosis induced by hypoxia/reoxygenation (H/R) *in vitro*. To this end, the rat cardiomyocyte H9C2 cell line was exposed to H/R and the roles of miR-21 and HIPK3 in regulating cell viability and apoptosis were evaluated by cell counting kit-8 assay, terminal-deoxynucleotidyl-transferase-mediated dUTP nick end labeling, and flow cytometry. Immunofluorescence and Western blotting were performed to detect the expression/phosphorylation of apoptosis-related proteins. miR‐21 expression was measured with quantitative real‐time polymerase chain reaction. The putative interaction between miR-21 and HIPK3 was evaluated using the luciferase reporter assay. Our results showed that (i) miR-21 overexpression or *HIPK3* down-regulation significantly attenuated H9C2 cells apoptosis after H/R, (ii) suppression of miR-21 expression promoted apoptosis, (iii) miR-21 overexpression inhibited HIPK3 expression, (iv) HIPK3 was the direct and main target of miR-21, (v) miR-21/HIPK3 formed part of a reciprocal, negative feedback loop, and (vi) *HIPK3* down-regulation decreased FAS-mediated apoptosis by inhibiting the phosphorylation of FADD, which subsequently inhibited the expression of BAX and cleaved caspase-3 and increased the expression of BCL2. Our study indicates that miR-21 attenuates FAS-mediated cardiomyocyte apoptosis by regulating HIPK3 expression, which could eventually have important clinical implications for patients with acute myocardial infarction.

## Introduction

Cardiovascular disease is the leading cause of death worldwide. It is usually attributed to the negative effects of acute myocardial ischemia–reperfusion (I/R) injury (IRI), for which there is still no effective prevention method [1]. Certain micro (mi)RNAs are implicated in IRI and their expression in cardiomyocytes changes markedly after this type of injury [2–4]. The role of miRNA-21 (miR-21) in cardiovascular disease has gained special attention [5,6]. Indeed, one study [7] identified miR-21 as one of the most important miRNAs implicated in numerous IRI-associated cardiovascular disease states. The authors of the present study used the online database miRDB to identify miR-21 target genes following IRI and found that it regulated several apoptotic genes. Of the 469 genes identified, the top target gene encoded FAS ligand (FASL) [7]. This finding suggests that miR-21 is associated with IRI-induced, FAS-mediated apoptosis.

Homeodomain interacting protein kinase 3 (HIPK3), a serine/threonine kinase, interacts with FAS-associated proteins [8,9], such as FAS-associating via death domain (FADD) and death-associated protein 6 (DAXX). Previous studies have shown that HIPK3 induces the phosphorylation of FADD at serine 194 [8] and promotes the apoptosis of tumor cells [10–13]. In addition, HIPK3 regulated cardiomyocyte apoptosis in a cell model of myocardial infarction [14]; however, there are few studies investigating the effect of HIPK3 on IRI-induced cardiomyocyte apoptosis.

Although both miR-21 and HIPK3 are associated with cardiomyocyte apoptosis, there is little information on how miR-21 and HIPK3 interact during myocardial IRI. Myocardial IRI can be simulated *in vitro* using myocardial hypoxia/reoxygenation (H/R). Thus, in the present study, we exposed H9C2 cells to H/R to determine the how miR‐21 and HIPK3 interacted to regulate apoptosis in the context of IRI.

## Materials and methods

### Cell culture, transfection, and H/R *in vitro*

The H9C2 embryonic rat cardiomyocytes cell line, derived from rat heart tissue, was provided by Cell Bank of Chinese Academy of Science (China). Cells were maintained in a complete medium, composed of Dulbecco’s Modified Eagle’s Medium (DMEM, Gibco, U.S.A.) supplemented with 10% fetal bovine serum (Gibco, U.S.A.) and 1% penicillin/streptomycin (Gibco, U.S.A.). Cells were randomized into eight groups: control (CTL), hypoxia/reoxygenation (H/R), H/R+miR-21 mimic (H/R+21mimic), H/R+miR-21 mimic negative control (H/R+21mimic NC), H/R+miR-21 inhibitor (H/R+21inhibitor), H/R+miR-21 inhibitor negative control (H/R+21inhibitor NC), H/R+small interfering (si)RNA-HIPK3 (H/R+si-HIPK3), and H/R+siRNA-HIPK3 negative control (H/R+si-NC).

Cells (except for those in the CTL and H/R groups) were transfected with the miR-21 mimic, miR-21 mimic negative control (NC), miR-21 inhibitor, miR-21 inhibitor NC, si-HIPK3, or si-HIPK3 NC (RiboBio, China) using Lipofectamine 2000 (Invitrogen, U.S.A.). The oligonucleotide sequences of miR-21 mimic, miR-21 mimic NC, miR-21 inhibitor, miR-21 inhibitor NC, and si-HIPK3 were as follows: miR-21 mimic, sense: 5′-UAGCUUAUCAGACUGAUGUUGA-3′ and anti-sense: 5′-UCAACAUCAGUCUGAUAAGCUA-3′; miR-21 mimic NC, sense: 5′-UUUGUACUACACAAAAGUACUG-3′, and anti-sense: 5′-CAGUACUUUUGUGUAGUA-CAAA-3′; miR-21 inhibitor, 5′-UCAACAUCAGUCUGAUAAGCUA-3′; miR-21 inhibitor NC, 5′-CAGUACUUUUGUGUAGUACAAA-3′; si-HIPK3, 5′-CCATTAGCAGTGACACTGA-3′. After substituting the complete medium with glucose-free DMEM (Gibco, U.S.A.), cells were incubated in a three‐gas incubator (Themor, U.S.A.) providing 94% N_2_, 1% O_2_, and 5% CO_2_ for 12 h to simulate a hypoxic state. After replacing the glucose-free DMEM with complete medium, cells were reoxygenated for 6 h at 37°C in a normoxic incubator with 95% air and 5% CO_2_. Cells in H/R group were incubated under the above-mentioned H/R conditions. Cells in CTL group were cultured in an incubator at 37°C, 5% CO_2_.

### Cell counting kit (CCK)-8 assay

Cells were seeded in 96-well culture plates at 6×10^4^ cells/ml. After undergoing transfection, the cells were exposed to H/R. About 10 µl CCK-8 reagent (Dojindo Molecular Technologies, Japan) was then added to each well. After 2 h, the optical density was determined spectrophotometrically at a wavelength of 450 nm.

### Lactate dehydrogenase (LDH) assay

Culture supernatant was collected. The LDH activity was measured with a LDH activity assay kit (Jiancheng Biotechnology, China), according to the manufacturer’s instructions.

### Terminal-deoxynucleotidyl-transferase-mediated dUTP nick end labeling (TUNEL)

Cells were seeded in 6-well culture plates at 3 × 10^5^ cells/ml. Cells were fixed with 4% paraformaldehyde. 0.5% Triton X-100 was used to increase the permeability of the cell membrane. Cells were then co-incubated with the TUNEL reagent (Elabscience, China) for 1 h. Nuclei were stained with 4',6′-diamidino-2-phenylindole (DAPI) for 5 min. Images were observed on a fluorescence microscope.

### Flow cytometry

Cells were harvested with trypsin (Gibco, U.S.A.), washed with phosphate-buffered saline, and suspended in 1× binding buffer. Next, 5 µl AnnexinV-PE (BD Biosciences, U.S.A.) and 5 µl 7‐amino‐actinomycin D (7AAD) (BD Biosciences, U.S.A.) were added to the cell suspension, followed by a 15–20 min incubation at room temperature in the dark. About 400 µl of 1× binding buffer was then added to each tube. Fluorescence was detected by a flow cytometer (BD Biosciences, U.S.A.) and apoptosis was quantified using Flowjo software. The total apoptosis rate was determined by AnnexinV-PE and 7AAD assay Q2 (early apoptosis) + Q3 (late apoptosis).

### Quantitative real‐time polymerase chain reaction (qRT-PCR)

Total RNA was extracted using the Trizol total RNA reagent (TIANGEN, China) and reverse-transcribed into cDNA using the FastKing RT Kit (TIANGEN, China). qRT-PCR was performed using the SuperReal PreMx Plus reagent (TIANGEN, China), with the following primers (GENERAL BIOL, China): *HIPK3* (NM_031144.3; 137 bp), forward: 5′-TCACAGAGGCTTGGAGACTG-3′ and reverse: 5′-ACAACATGTGCGATGCCTAC-3′; beta-actin (NM_031787.2; 173 bp), forward: 5′-CACCATGTACCCAGGCATTG-3′ and reverse: 5′-CCTGCTTGCTGATCCACATC-3′. Reaction conditions were as follows: pre-denaturation at 95 °C for 15 min, followed by 40 cycles of denaturation at 95 °C for 10 s, and annealing and extension at 60°C for 32 s. Beta-actin served as an internal control. The miRcute miRNA Isolation Kit was used to extract miRNA and cDNA was generated with miRcute Plus miRNA First-Strand cDNA Kit (TIANGEN, China). qRT‐PCR was carried out using the miRcute Plus miRNA qPCR Kit (TIANGEN, China) using the following reaction conditions: pre-denaturation at 95°C for 15 min, followed by 45 cycles of denaturation at 94°C for 20 s, and annealing and extension at 60°C for 34 s. Data were normalized to U6 spliceosomal RNA. The upstream and downstream miR-21 and U6 primers were designed by RiboBio Corporation (China).

### Western blotting

Total protein was obtained with lysis buffer and was then separated by sodium dodecyl sulfate‐polyacrylamide gel electrophoresis (SDS‐PAGE) and transferred to polyvinylidene difluoride (PVDF) membranes. After blocking for 60 min in 5% skimmed milk, membranes were incubated at 4°C overnight with antibodies targeting HIPK3 (ab72538, Abcam, U.K.; 1:1,000 dilution), FADD (14906-1-AP, Proteintech, China; 1:7,500 dilution,), phospho-FADD Ser 194 (DF2996 Affinity, China; 1:1,200 dilution), BAX (ab32503, Abcam, U.K.; 1:8,000 dilution), BCL‐2 (ab59348, Abcam, U.K.; 1:1,000 dilution), caspase-3 (#9662, Cell Signaling Technology, U.S.A.; 1:1,000 dilution), and bata Actin (AF7018, Affinity, China; 1:15,000 dilution). Membranes were then washed with tris‐buffered saline Tween (TBST) and incubated with a horseradish peroxidase (HRP)‐conjugated goat anti‐rabbit secondary antibody (S0001, Affinity, China; 1:8,000 dilution) for 1 h at room temperature. Protein bands were visualized with enhanced chemiluminescence detection reagents and quantified by densitometry using ImageJ software.

### Immunofluorescence

Cells were fixed with 4% paraformaldehyde for 20 min and then permeabilized by incubating with 150 μl 0.5% Triton X-100 for 15 min at room temperature. After blocking for 60 min in normal goat serum (ZGGB-BIO, China), cells were incubated overnight with an anti-HIPK3 antibody (ab72538, Abcam, U.K.; 1:150 dilution) diluted in 5% bovine serum albumin at 4°C. The next day, cells were incubated for 60 min with a goat anti‐rabbit secondary antibody (A22220, Abbkine, U.S.A.; 1:200 dilution), counterstained with DAPI, and imaged on a fluorescence microscope.

### Luciferase reporter assay

The luciferase reporter assay was performed using a psiCHECK™ -2 vector (Promega, U.S.A.), which contained either a wildtype or a mutant version of the HIPK3 3′‐UTR (HIPK3‐WT‐3′‐UTR or HIPK3‐MUT‐3′‐UTR, respectively). In the mutant HIPK3 vectors, the 5′-ATAAGCTA-3′ wildtype miR-21 binding site was replaced with the 5′-CTCCGATC-3′ miR-21 binding site. H9C2 cells were divided into four groups. HIPK3-WT+NC cells were co-transfected with miR-21 mimic NC and psiCHECK™ -2 vector containing HIPK3-WT-3′-UTR; HIPK3-WT+21mimic cells were co-transfected with miR-21 mimic and psiCHECK™-2 vector containing HIPK3-WT-3′-UTR; HIPK3-MUT+NC cells were co-transfected with miR-21 mimic NC and psiCHECK™-2 vector containing HIPK3-MUT-3′-UTR; and HIPK3-MUT+21mimic cells were co-transfected with miR-21 mimic and psiCHECK™-2 vector containing HIPK3-MUT-3′-UTR. Cells were seeded into 96-well plates at 1 × 10^4^ cells/well and co-transfected for 24 h. Luciferase activity was detected with a luciferase reporter assay kit (Promega, U.S.A.). The ratio of *Renilla* luminescence to firefly luminescence was calculated to determine relative luciferase activity.

### Statistical analysis

Data were presented as means±standard deviation (SD) and analyzed using SPSS 25.0 software. One-way ANOVA and Welch variance analysis were used for comparisons. *P*-values<0.05 were considered as a measure of statistical significance.

## Results

### Effect of miR-21 levels on H9C2 cell viability and LDH activity after H/R

**Figure 1:**
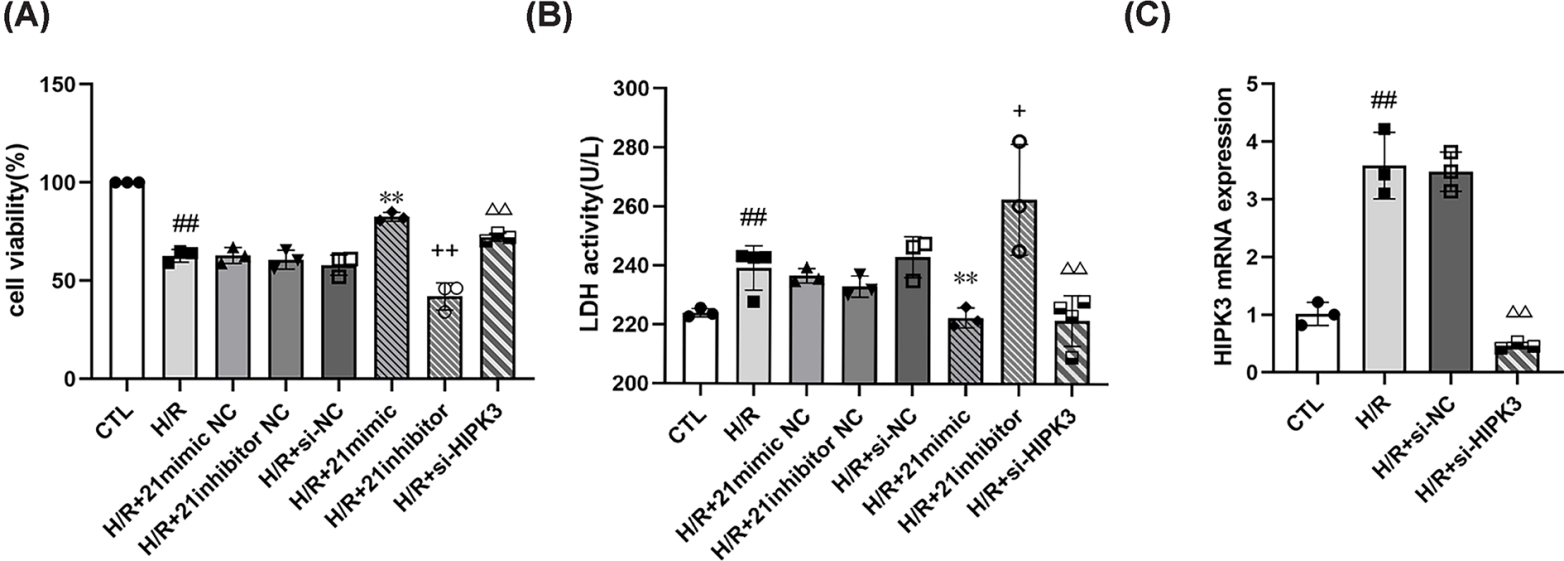
MiR-21 overexpression and *HIPK3* down-regulation increase cell viability and decrease LDH activity (**A**) The cell viability relative to CTL: miR-21 overexpression or *HIPK3* down-regulation increased cell viability while miR-21 suppression decreased cell viability (*n*=3). (**B**) LDH activity: miR-21 overexpression or *HIPK3* down-regulation decreased LDH activity while miR-21 suppression increased LDH activity (*n*=3 or 4). (**C**) *HIPK3* mRNA expression relative to CTL (*n*=3). Data were presented as mean ± SD; ^##^*P*<0.01 vs. CTL; **P<0.01 vs. H/R+ 21mimic NC; ^+^*P*<0.05 vs. H/R+ 21inhibitor NC; ^++^*P*<0.01 vs. H/R+ 21inhibitor NC; ^ΔΔ^*P*<0.01 vs. H/R+ si-NC.

We found that miR-21 greatly affected H9C2 cell viability and LDH activity after H/R (Figure 1A,B). miR-21 overexpression increased cell viability and decreased the release of LDH, while suppression of miR-21 expression had the opposite effect. These results indicate miR-21 greatly increases cell viability and decreases cell injury after H/R.

### Down-regulation of *HIPK3* increases H9C2 cell viability and decreased LDH activity after H/R

We confirmed that the expression of *HIPK3* mRNA was effectively down-regulated following the transfection of H9C2 cells with si-HIPK3 (Figure 1C). Cells transfected with si-HIPK3 exhibited increased cell viability (Figure 1A) and decreased LDH activity than H/R+si-NC group (Figure 1B). These findings indicate that *HIPK3* down-regulation greatly increases cell viability and decreases cell injury after H/R.

### Effect of miR-21 levels on H9C2 cell apoptosis after H/R

**Figure 2:**
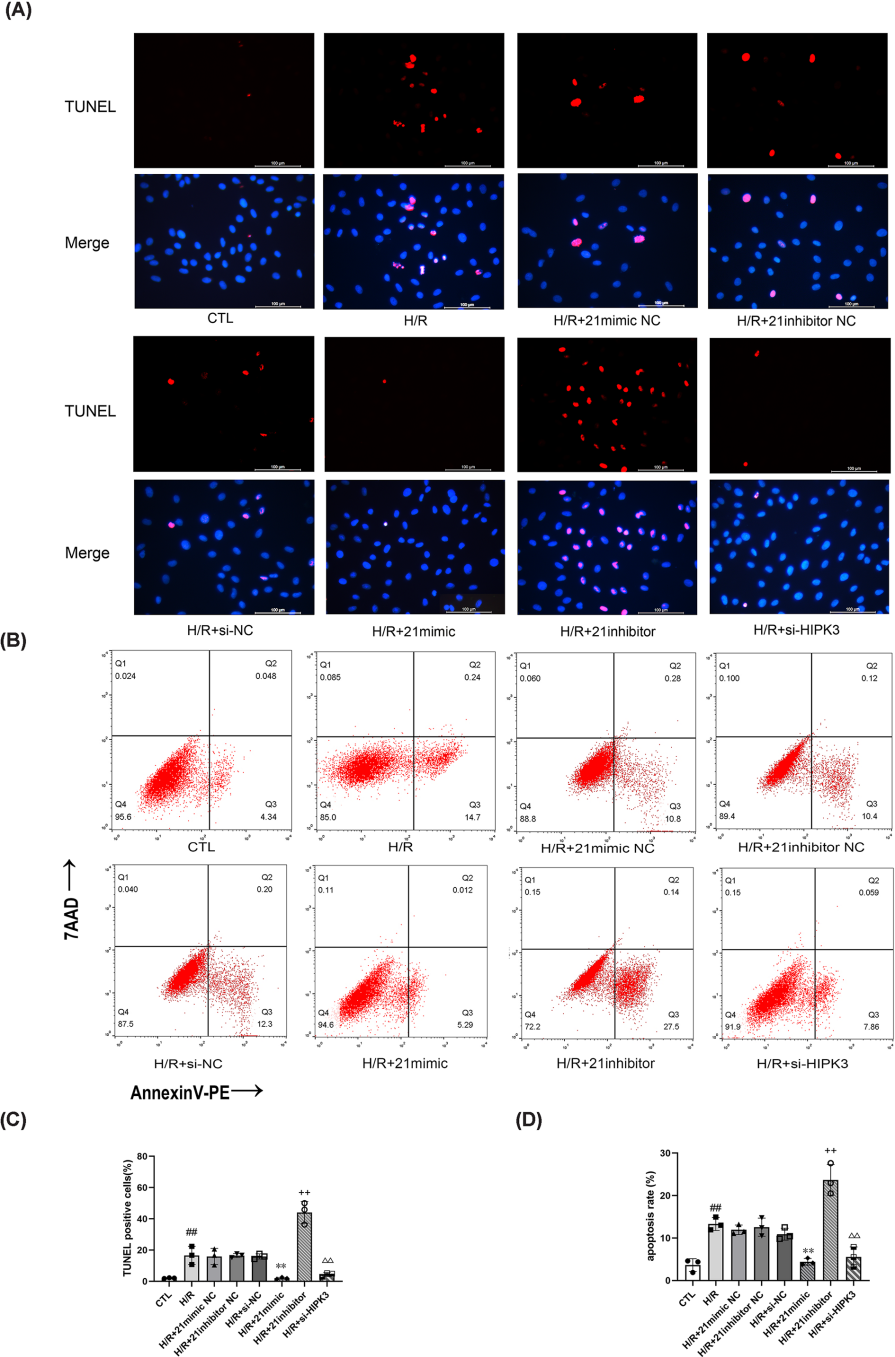
MiR-21 overexpression and *HIPK3* down-regulation suppress apoptosis (**A**) TUNEL staining showed miR-21 overexpression or *HIPK3* down-regulation significantly reduced TUNEL positive cells, and miR-21 suppression increased TUNEL positive cells (×400 magnification). (**B**) Representative images of flow cytometry: miR-21 overexpression or *HIPK3* down-regulation inhibited apoptosis, and miR-21 suppression promoted apoptosis. (**C**) Quantitative analysis of TUNEL positive cells (*n*=3). (**D**) Rates of apoptosis cells detected by flow cytometry (*n*=3). Data are presented as mean ± SD; ^##^*P*<0.01 vs. CTL; **P<0.01 vs. H/R+21mimic NC; ^++^*P* <0.01 vs. H/R+ 21inhibitor NC; ^ΔΔ^*P*<0.01 vs. H/R+si-NC.

**Figure 3:**
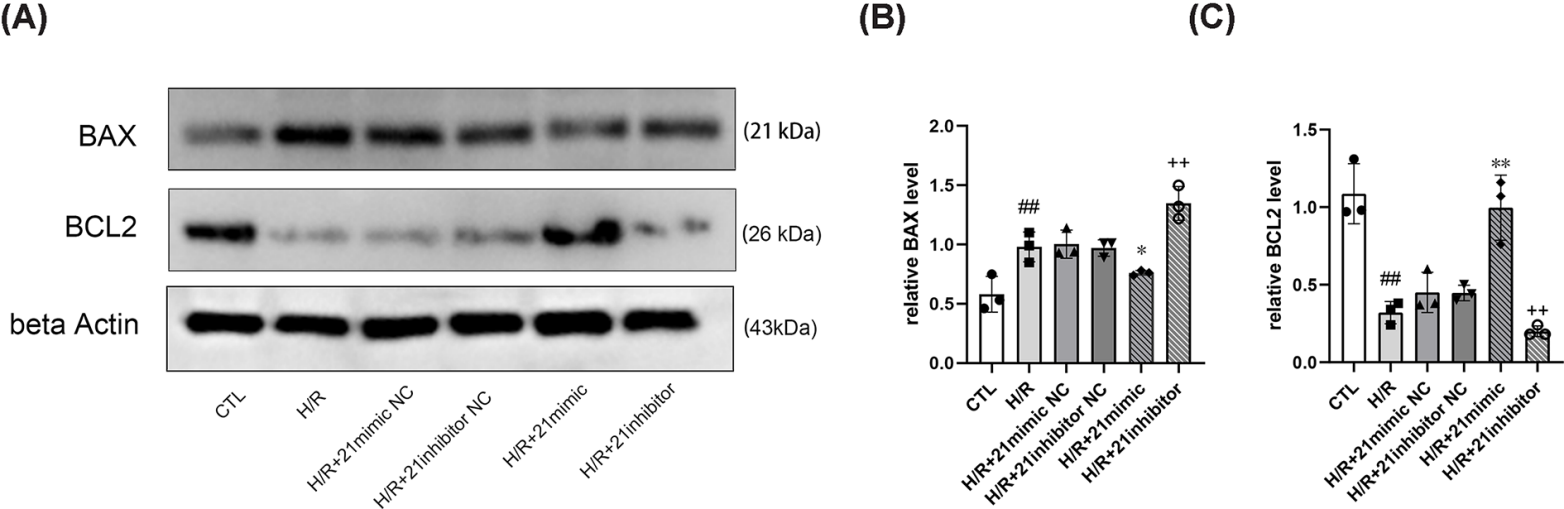
MiR-21 affects apoptosis-related proteins (**A**) Representative images of Western blot analysis. (**B**) and (**C**) Statistical analysis of protein levels: miR-21 overexpression decreased BAX expression and increased BCL2 expression, and miR-21 suppression increased BAX expression and decreased BCL2 expression. The gray values of bands were normalized to that of beta Actin (*n*=3). Data are presented as mean ± SD; ^##^*P*<0.01 vs. CTL; *P<0.05 vs. H/R+ 21mimic NC; **P<0.01 vs. H/R+ 21mimic NC; ^++^*P*<0.01 vs. H/R+ 21inhibitor NC.

We found that apoptosis and BAX expression increased and BCL2 expression decreased after H/R. However, miR-21 overexpression decreased apoptosis and BAX expression and increased BCL2 expression, while the suppression of miR-21 expression had the opposite effect (Figures 2A–D and 3A–C). These results indicate that miR-21 has a protective effect by limiting apoptosis after H/R.

### *HIPK3* down-regulation suppresses H9C2 cell apoptosis after H/R

Transfection of H9C2 cells with si-HIPK3 significantly decreased their rate of apoptosis (Figure 2A–D). This result indicates that *HIPK3* down-regulation inhibits apoptosis after H/R.

### miR-21/HIPK3 form part of a reciprocal, negative feedback loop

**Figure 4:**
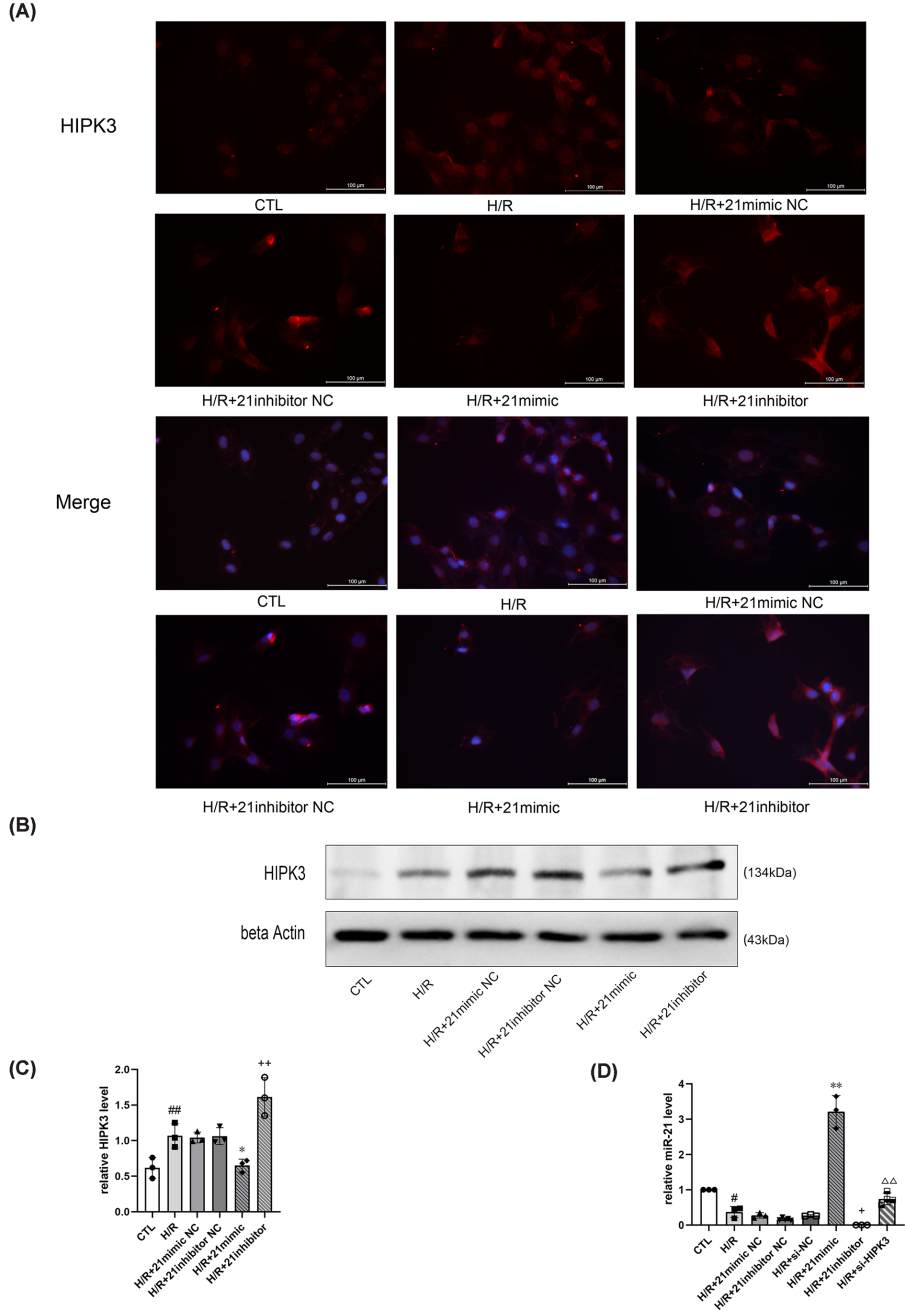
MiR-21 interacts with HIPK3 (**A**) Representative immunofluorescence images of HIPK3 expression levels (×400 magnification). (**B**) Representative images of Western blot analysis. (**C**) Statistical analysis of HIPK3 levels: miR-21 overexpression inhibited HIPK3 expression, and miR-21 suppression up-regulated HIPK3 expression. The gray values of bands were normalized to that of beta Actin (*n=*3). (**D**) MiR-21 expression relative to CTL: transfecting with si-HIPK3 increased miR-21 expression, and transfecting with miR-21 mimic increased miR-21 expression while transfecting with miR-21 inhibitor decreased miR-21 expression (*n*=3–5). Data are presented as mean ± SD; ^#^*P*<0.05 vs. CTL; ^##^*P*<0.01 vs. CTL; *P<0.05 vs. H/R+ 21mimic NC; **P<0.01 vs. H/R+21mimic NC; ^+^*P*<0.05 vs. H/R+21inhibitor NC; ^++^*P*<0.01 vs. H/R+ 21inhibitor NC; ^ΔΔ^*P*<0.01 vs. H/R+ si-NC.

We found that miR-21 overexpression inhibited HIPK3 expression, while suppression of miR-21 expression had the opposite effect (Figure 4A–C). Meanwhile, *HIPK3* down-regulation increased miR-21 expression (Figure 4D). These results indicate that miR-21 interacts with HIPK3, forming part of a reciprocal negative feedback loop.

### HIPK3 is a direct and main target of miR-21

**Figure 5:**
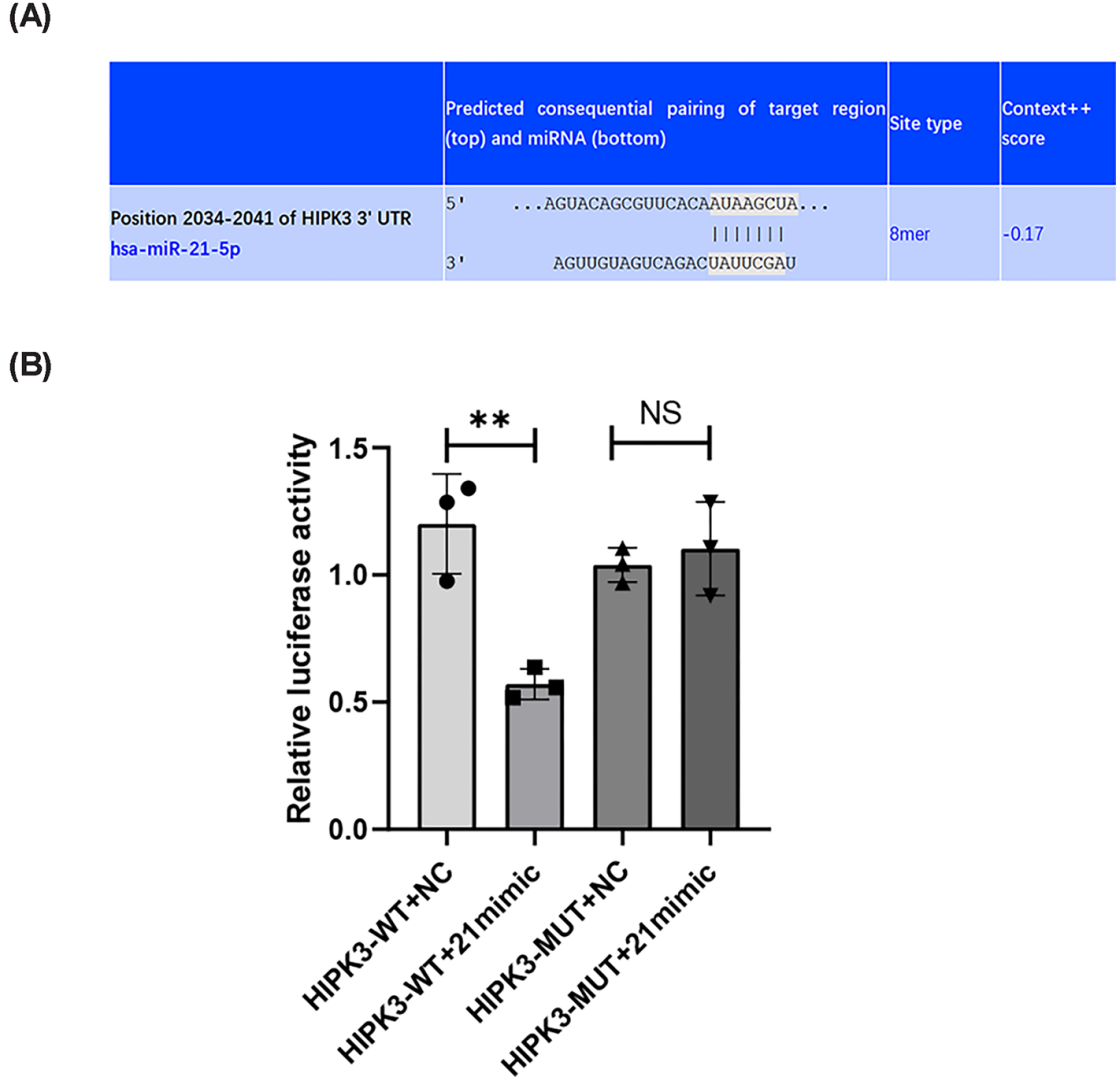
MiR‐21‐5p directly binds to 3′‐UTR of HIPK3 (**A**) The binding site of miR‐21 with HIPK3 3′‐UTR was predicted in the TargetScan 7.2 website. (**B**) Luciferase activity of HIPK3‐WT‐3′‐UTR or HIPK3‐MUT‐3′‐UTR after cotransfecting miR-21 mimic was detected by dual luciferase reporter assay. Luciferase activity of H9C2 cells cotransfected with miR-21 mimic and psiCHECK™-2 vector containing HIPK3-WT-3′-UTR significantly decreased (*n*=3). Data are presented as mean ± SD; **P<0.01; NS, no significance.

TargetScan 7.2 predicted that HIPK3 was a direct target of miR-21 (Figure 5A). We found that the relative luciferase activity of H9C2 cells co-transfected with miR-21 mimic and psiCHECK™-2 vector containing HIPK3-WT-3′-UTR was significantly lower than that of the HIPK3-WT+NC group; however, this effect was not observed in cells co-transfected with miR-21 mimic and psiCHECK™-2 vector containing HIPK3-MUT-3′‐UTR (Figure 5B). The results prove that miR-21 directly targets HIPK3. After co-transfecting H9C2 cells with si-HIPK3 and miR-21 inhibitor, the effect of the miR-21 inhibitor on apoptosis and cell viability was significantly diminished (Supplementary Figure S1A–C). This finding confirms that HIPK3 is the main target of miR-21.

### Effect of HIPK3 on FAS-mediated apoptosis

**Figure 6:**
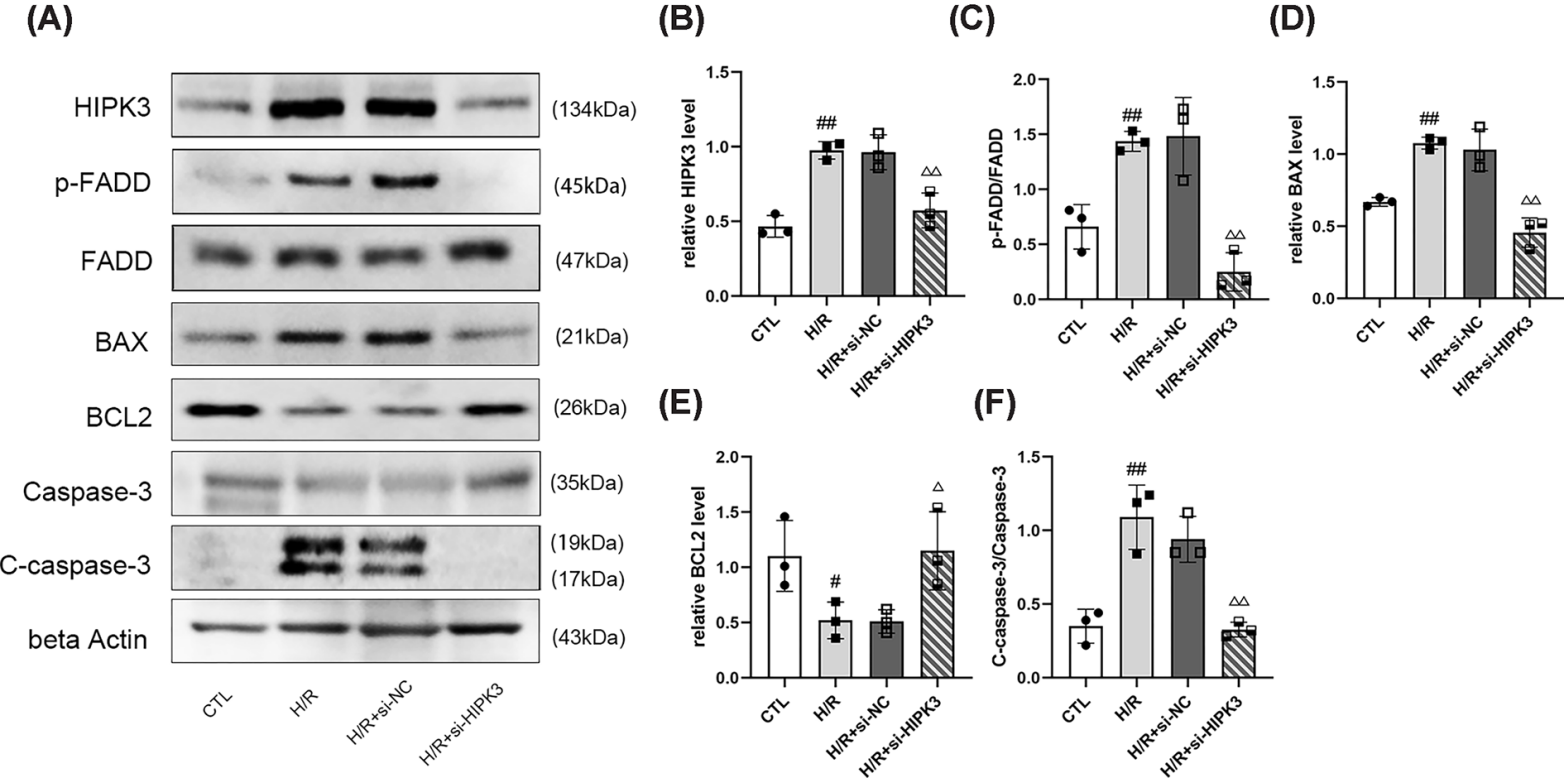
*HIPK3* down-regulation affects the expression of proteins relative to Fas-mediated apoptosis (**A**) Representative images of Western blot analysis. (**B–F**) Statistical analysis of protein levels: *HIPK3* down-regulation decreased the phosphorylation of FADD and the expression of BAX and C-Caspase-3, and increased the expression of BCL2 (*n*=3). In panels B,D,E, the gray values of bands were normalized to that of beta Actin. C-Caspase-3, cleaved caspase-3; p-FADD, phosphorylated FADD. Data are presented as mean ± SD; ^#^*P*<0.05 vs. CTL; ^##^*P*<0.01 vs. CTL; ^Δ^*P*<0.05 vs. H/R+ si-NC; ^ΔΔ^*P*<0.01 vs. H/R+ si-NC.

Down-regulation of *HIPK3* decreased FAS-mediated apoptosis by inhibiting the phosphorylation of FADD. The reduced phosphorylation of FADD inhibited the expression of BAX and cleaved caspase-3 (C-caspase-3), while promoting the expression of BCL2 (Figure 6A–F).

## Discussion

miR-21 is an abundant miRNA in cardiomyocytes and is tightly associated with myocardial IRI [5,6]. The up-regulation of miR-21 has been confirmed in various pathological conditions [15–19]; however, several studies have reported the down-regulation of miR-21 in myocardial IRI [20,21]. Consistent with the results of these myocardial IRI studies, we also showed that miR-21 was down-regulated after H/R in H9C2 cells. Since apoptosis is a common pathological phenomenon in the I/R-induced death of cardiomyocytes, it is often used to assess the extent of IRI [22]. Sayed et al. [21] found that the overexpression of miR-21 in mouse hearts with IRI pathology resulted in a smaller infarct size and a lower cardiomyocyte apoptosis rate. Moreover, Cheng et al. [23] found that ischemic preconditioning (IP)-induced cardiac protection against I/R was eliminated, while cardiomyocyte apoptosis rate was increased, in miR-21-deficient rats. Our study showed that miR-21 overexpression attenuated cardiomyocyte apoptosis after H/R, while the inhibition of miR-21 expression had the opposite effect, which suggests that miR-21 plays an anti-apoptotic role in cardiomyocytes after H/R.

HIPK3 is aberrantly expressed in malignant tumors and is associated with apoptosis [10–13]. Researchers confirmed that FADD is a substrate of HIPK3, and that HIPK3 affects the phosphorylation of FADD at serine 194 [8,10]; this modification is the key to FAS-mediated apoptosis and the subsequent nuclear translocation of FADD [24]. In addition, FAS is required for the stabilization of the HIPK3-FADD complex [8]. Moreover, the phosphorylated FADD enhances the activation of MEK kinase 1 (MEKK1) and c-jun NH_2_-terminal kinase 1 (JNK1), which further promotes apoptosis [25]. In our study, *HIPK3* down-regulation decreased FADD phosphorylation and cardiomyocyte apoptosis after H/R; however, whether the phosphorylation of FADD drives cardiomyocyte apoptosis by enhancing MEKK1 and JNK1 activation remains unclear.

Both miR-21 and HIPK3 play a role in cardiomyocyte apoptosis; however, it was unclear whether they interacted with each other. Our study showed that miR-21 interacted with HIPK3 to protect cardiomyocytes against H/R-induced apoptosis, likely via the miR-21-mediated inhibition of HIPK3 expression. Specifically, we showed that (i) H/R decreased the expression of miR-21 and concomitantly increased that of HIPK3; (ii) miR-21 overexpression reduced HIPK3 expression and apoptosis, while suppressing miR-21 expression had the opposite effect; (iii) *HIPK3* down-regulation increased miR-21 expression and reduced apoptosis; (iv) co-transfecting cardiomyocytes with si-HIPK3 and a miR-21 inhibitor significantly decreased the pro-apoptotic effect of the miR-21 inhibitor; (v) miR-21-5p directly targeted the 3′-UTR of *HIPK3* mRNA.

We found that miR-21 and HIPK3 functioned as part of a reciprocal, negative feedback loop, whereby, miR-21 overexpression inhibited HIPK3 expression, while *HIPK3* downregulation increased miR-21 expression. We speculate that this feedback loop helps maintain high miR-21 levels in H9C2 cells during H/R and enables it to exert its anti-apoptotic effect; this phenomenon has also been found in other studies of miR-21. For instance, Liang et al. [26] reported a reciprocal miR-21/transforming growth factor β-receptor III (TGFβRIII) loop in cardiac fibroblasts, whereby miR-21 up-regulation resulted in the down-regulation of *TGFβRIII*, which increased TGF-β1 expression; conversely, the increase in TGF-β1 expression up-regulated the expression of miR-21. Sun et al. [27] also reported a reciprocal, negative feedback loop involving miR-21/programmed cell death 4 (PDCD4)/activation protein-1 (AP-1) in renal fibrogenesis, whereby miR-21-mediated PDCD4 inhibition enhanced AP-1 activity, and AP-1 in turn promoted miR-21 transcription. Because reciprocal, negative feedback loops involving miR-21 have been identified in a variety of diseases, we believe that such a mechanism is crucial for the biological function of miR-21.

miR-21 regulates multiple mRNAs by altering their transcriptional stability or impacting on their translation [28]. For instance, the overexpression of miR-21 inhibited the expression of phosphatase and tensin homolog deleted on chromosome 10 (PTEN) in a mouse model of IRI [29] and PDCD4 expression in an IRI rat model and H/R-exposed H9C2 cells, which subsequently inhibited apoptosis [30]. However, by co-transfecting H9C2 cells with si-HIPK3 and miR-21 inhibitor, we observed that *HIPK3* down-regulation effectively reversed the pro-apoptotic action of the miR-21 inhibitor after H/R. Therefore, we believe that miR-21 mainly targets HIPK3 in H/R H9C2 cells but cannot exclude the possibility that other miR-21 targets may also play a role.

In conclusion, we confirmed that miR-21 attenuated cardiomyocyte apoptosis after H/R by inhibiting HIPK3 expression. Reduced HIPK3 levels in turn inhibited FAS-mediated apoptosis by preventing FADD phosphorylation. We also confirmed that miR-21 directly and predominantly targets HIPK3 and forms part of a reciprocal, negative miR-21/HIPK3 feedback loop.

The data from the present study provide a potential therapeutic approach for preventing IRI-associated apoptosis, which could be applied in a clinical setting to improve the prognosis of patients with acute myocardial infarction. However, because all of our data were generated using H9C2 cells *in vitro*, they will need to be validated in animal IRI models and clinical trials.

## Supplementary Material

Supplementary Figures S1

## Data Availability

The datasets used and/or analyzed during the current study are available from the corresponding author on reasonable request.

## Abbreviations

AP-1: activation protein-1
C-caspase-3: Cleaved caspase-3
Daxx: death-associated protein 6
FADD: Fas-associating via death domain
FASL: FAS ligand
JNK1: c-jun NH_2_-terminal kinase 1
HIPK3: homeodomain interacting protein kinase 3
HIPK3‐MUT‐3′‐UTR: HIPK3‐mutant3′‐UTR
HIPK3‐WT‐3′‐UTR: HIPK3‐wild‐type‐3′‐UTR
H/R: hypoxia/reoxygenation
IP: ischemic preconditioning
IRI: ischemic reperfusion injury
I/R: ischemia–reperfusion
LDH: lactate dehydrogenase
MEKK1: MEK kinase 1
mi: micro
miR‐21: microRNA-21
NC: negative control
PDCD4: programmed cell death 4
PTEN: phosphatase and tensin homolog
p-FADD: phosphorylated FADD
TGFβRIII: transforming growth factor β receptor III
TUNEL: terminal-deoxynucleotidyl-transferase-mediated dUTP nick end labeling
