# Supplementary material for: MiR-21 attenuates FAS-mediated cardiomyocyte apoptosis by regulating HIPK3 expression
Source: Biosci Rep. 2023 Sep 5;43(9):BSR20230014. doi: 10.1042/BSR20230014 (PMC10500225; doi:10.1042/BSR20230014)
Supplement: Supplementary Figures S1 [file BSR-2023-0014_supp.pdf]

Supplementary materials

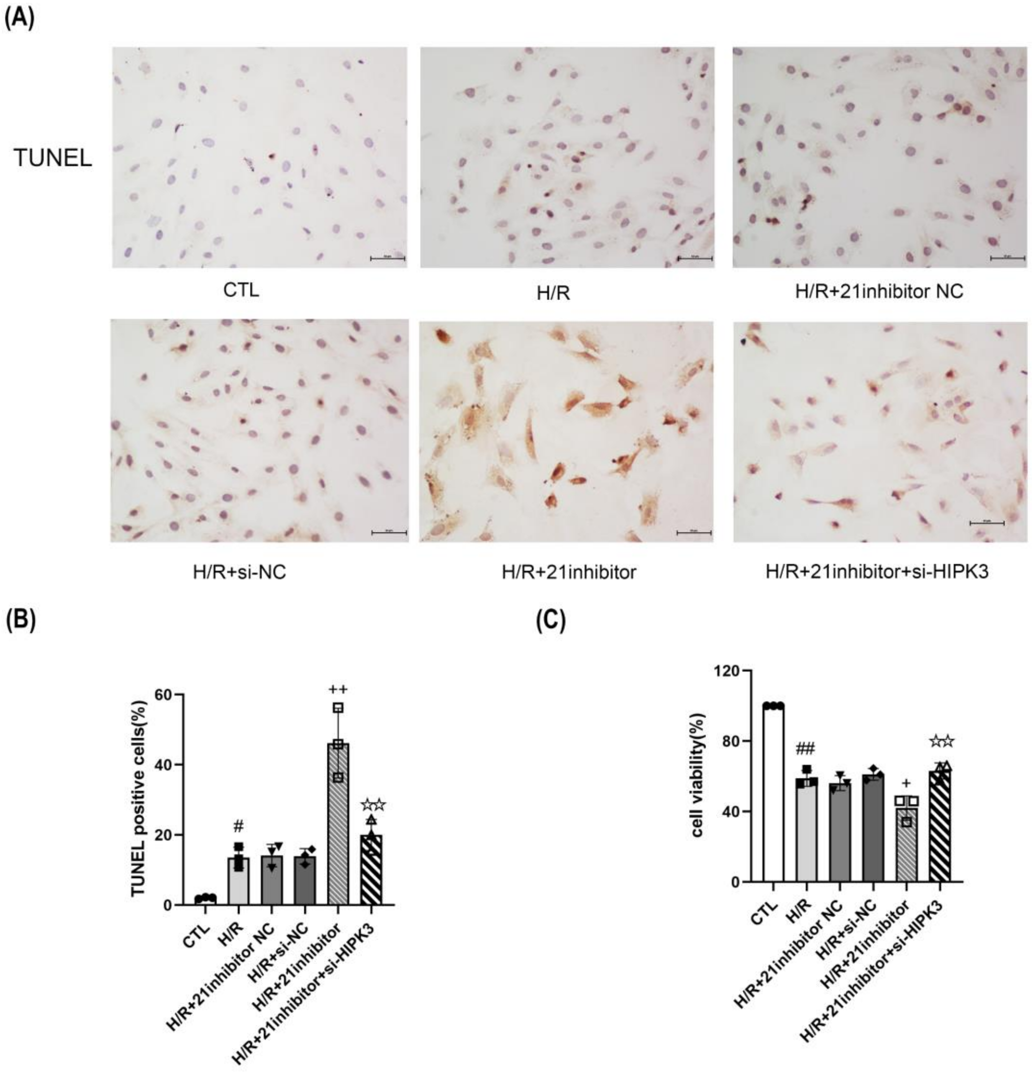

Supplementary Figure S1 HIPK3 is a main target of miR-21. (A) TUNEL staining showed *HIPK3* downregulation significantly reduced apoptosis induced by miR-21 inhibitor (×200 magnification). (B) Quantitative analysis of TUNEL positive cells (n=3). (C) The cell viability relative to CTL: *HIPK3* downregulation significantly increased cell viability which was reduced by miR-21 inhibitor (n=3). Cells in H/R+21inhibitor+si-HIPK3 were cotranfected with miR-21 inhibitor and si-HIPK3 before H/R. Data are presented as mean±SD; $^{\#\#}$ *P* <0.01 vs CTL; $^{+}$ *P* <0.05 vs H/R+ 21inhibitor NC; $^{++}$ *P* <0.01 vs H/R+ 21inhibitor NC; $^{☆☆}$ *P* <0.01 vs H/R+21inhibitor.
